# CT-guided iodine-125 brachytherapy as salvage therapy for local-regional recurrent breast cancer

**DOI:** 10.3389/fonc.2023.1171813

**Published:** 2023-08-18

**Authors:** Juan Wang, Xiaojing Chang, Ke Xu, Yansong Liang, Jinxin Zhao, Zezhou Liu, Hongtao Zhang

**Affiliations:** ^1^ Department of Oncology, Hebei General Hospital, Shijiazhuang, Hebei, China; ^2^ Department of Radiotherapy, The Second Hospital of Hebei Medical University, Shijiazhuang, Hebei, China

**Keywords:** iodine-125 seed, brachytherapy, computed tomography guidance, breast cancer, salvage therapy, recurrence

## Abstract

**Background:**

The treatment of local–regional recurrent breast cancer (BC) after external beam radiotherapy is challenging. We aim to evaluate the effectiveness and safety of computed tomography (CT)–guided percutaneous iodine-125 brachytherapy for local recurrent BC.

**Methods:**

We retrospectively analyzed 15 patients with local recurrent BC treated with CT-guided interstitial implantation of iodine-125 seeds. Regular contrast-enhanced CT was conducted to evaluate the tumor response. Follow-up survival, quality of life, and adverse events were analyzed.

**Results:**

Among the 15 patients, five were elderly patients (older than 80 years) and six were complicated with chronic underlying diseases. The median number of ^125^I seeds implantation was 33 (range: 20–130) with median dose 90 (D90, the minimum dose covering 90% of the target volume) of 108 Gy (range: 60–120 Gy). There was no significant difference in D90, V100 (the volume of the target receiving 100% of the prescription dose), and V150 (the volume of the target receiving 150% of the prescription dose) before and after operation (*p* > 0.05). The median follow-up was 14 months (range: 6–18 months). Six months after operation, the ORR was 66.7% (10/15) and the LCR was 93.3% (14/15). The 6- and 12-month survival rates were 100 and 41.6%, respectively, and the median survival time was 12.5 months. PS score decreased from 1.53 ± 0.81 to 0.53 ± 0.49. The pain score decreased from 2.87 ± 1.67 before operation to 1.07 ± 1.18 after operation, and the differences were statistically significant (*p*< 0.05). No severe complications occurred.

**Conclusions:**

CT-guided iodine-125 brachytherapy provided a safe and effective choice for recurrent BC with significant local therapeutic effects and minor complications, especially for elderly patients with chronic underlying disease and those who were not eligible for surgical resection and had failed to benefit from systemic therapy.

## Introduction

Breast cancer (BC) is the most common malignancy in women, and the latest data showed that BC had been the most commonly diagnosed cancer worldwide and the leading cause of cancer death worldwide in women in 2020 (1). In China, the latest data from the National Cancer Center demonstrate that its morbidity ranks the fifth (2). To date, external beam radiotherapy (EBRT), which could reduce the local recurrence rate and prolong the progression-free survival (PFS) of BC patients, has become the standard treatment after modified radical mastectomy and Breast-Sparing Surgery (3, 4). Unfortunately, approximately 5–30% of patients with BC who underwent surgical resection, chemoradiotherapy, endocrine, and/or target therapy presented with local recurrence (5, 6). Recurrence is considered the major prognostic factor for BC patients; to data, the treatment is challenging, given their specific location and the modality of previous multiple therapies, such as surgery, chemotherapy, and radiation. Currently, there is no standard treatment approach for recurrent BC. Iodine-125 (^125^I) seed implantation with low-dose rate (LDR), is a type of brachytherapy radiation technique that is characterized by extremely a sharp drop-fall off dose achieving an accurate dose delivery and sparing the normal tissues that EBRT could never achieve within treated lesions, has been considered as a standard therapy for localized prostate carcinoma (7, 8). To date, an increasing number of studies showed adequate efficacy of ^125^I brachytherapy in many solid tumors (9–11). However, there are a few reports regarding ^125^I brachytherapy for recurrent BC. Therefore, we conducted this study to evaluate the effectiveness and safety of ^125^I brachytherapy for the treatment of recurrent BC patients to provide an alternative therapy for these difficult cases.

## Material and methods

### Patients

A total of 15 patients diagnosed with chest wall recurrence BC (total of 15 lesions) who underwent interstitial ^125^I brachytherapy as a salvage treatment with a CT-guided coplanar template-assisted technique between December 2011 and May 2022 in our hospital were retrospectively collected. Among these patients, one was man, 14 were women, while five of these 15 patients were elderly patients (older than 80 years), six of these 15 patients were complicated with chronic underlying diseases (such as hypertension, diabetes, coronary heart disease), and one of these patients with local huge ulcer at the chest wall recurrent lesion. All of these patients had previously received surgical resection, chemotherapy, and external beam radiation. Baseline clinical characteristics of these patients were shown in **Table 1**. This study was approved by the institutional review board of our hospital, and written informed consent was obtained from all of the patients. The inclusion criteria in our study were all follows: All cases were pathologically diagnosed, and inability to tolerate or refuse surgical resection and external radiotherapy; all patients agreed to receive ^125^I brachytherapy as a salvage treatment; Karnofsky performance status (KPS) ≥ 70; blood routine examination and coagulation function were normal; and expected survival ≥ 3 months.

**Table 1:** Baseline clinical characteristics of patients.

No.	Sex	Age	Pathology type	Stage	Pre/post-operative treatment	Pre-operative edema	Underlying disease
1	F	59	IDC	IIIA	S+CTx/T	Yes	Hypertension
2	F	65	Sarcoma	IIIC	S+CTx/CTx	N	N
3	F	63	IDC	IIIC	S+RT/CTx	N	N
4	F	83	IDC	IV	None	N	Diabetes
5	F	84	IDC	IV	H	N	N
6	F	52	IDC	IIA	S+CTx/CTx	N	N
7	F	89	IDC	IIIB	None	Yes	Coronary heart disease
8	F	89	IDC	IV	None	N	Hypertension
9	F	53	IDC	IIB	CTx/H	N	Hypertension
10	F	61	IDC	IIIC	S+CTx/CTx	Yes	Diabetes
11	M	68	IDC	IIB	S+CTx/CTx	N	N
12	F	78	IDC	IIB	S+CTx/CTx	N	N
13	F	86	IDC	IV	S+CTx/T	N	Local ulcer
14	F	58	IDC	IV	S/RT+CTx	N	N
15	F	63	IDC	IV	RT+CTx/none	N	N

IDC, invasive ductal cancer; S, surgery; RT, radiotherapy; CTx, chemotherapy; H, endocrine therapy; T, targeted therapy; N, none.

The exclusion criteria were all follows: severe organ dysfunction; coagulation dysfunction, anticoagulant therapy should be stopped at least 5–7 days before implantation; poor general condition or cachexia; the interval from last radiotherapy was less than 3 months; no CT and other imaging data after ^125^I seed implantation.

### Instrument and equipment

The 18-G implantation needles and seed implant applicator were provided from Mick Radio-Nuclear company, USA. The ^125^I seeds (ZHIBO Bio-Medical Tech Ltd. Beijing, China) used in this study were cylindrical and 4.5-mm long with a diameter of 0.8 mm. The seed activity was 0.3–0.7 mCi and had a half-life of 59.4 days. The γ-ray energy was 27–35 KeV. The tissue half-value layer was 1.7 cm. About 93–97% of the energy of the seed was delivered into the tumor after 8–10 months.

A treatment planning system (TPS) (Panther Brachy version 5.0 TPS, Prowess Inc., Concord, CA, USA) was used to develop a treatment plan. The RM-905a radioactivity meter was provided by the Chinese Institute of Metrology. PET-CT (Discovery CT750 HD, GE, USA) was used to scan images.

### Methods of ^125^I seeds implantation

Preoperative preparation: Enhanced CT scan (slice thickness 5 mm) was performed 1 week before surgery, and the images were transmitted to TPS to make preoperative plan. The dose of ^125^I seed implantation was prescribed as D90, V100, and V150, which encompassed the planning treatment volume (PTV). PTV included the entire gross tumor volume (GTV) and 0.5- to 1.0-cm margins.

Intraoperative operation: The position was consistent with the preoperative design, local anesthesia with 1% lidocaine, CT, and ultrasound localization scan to determine the puncture point, angle, and depth of the body surface. According to the preoperative plan, the center was sparsely implanted with particles spaced between 0.5–1.0 cm. CT scan was performed immediately after operation to observe the spatial distribution of particles, and the existing dosimetric cold area was re-implanted.

Postoperative treatment: The quality of postoperative CT scan images was verified by transferring them into TPS. The actual dose to the tumor was obtained according to the dose-volume histogram (DVH).

### Treatment of post ^125^I seed implantation

After ^125^I seed implantation, seven of these 15 patients received four to six cycles of chemotherapy, two patients just received endocrine therapy, two patients received target therapy, and the other four patients received no antitumor drug therapy. Chemotherapy and endocrine therapy were performed according to the latest guidelines of the National Comprehensive Cancer Network (NCCN).

### Follow-up and clinical efficacy evaluation

Follow-up CT was conducted at 1 month after the procedure, 3 months after the procedure, and then every 3 months. The primary end points were the objective response rate (ORR) and tumor local control rate (LCR). The secondary end points included the clinical benefit response (CBR), overall survival (OS), and incidence of adverse events. The ORR was defined as the proportion of patients achieving complete response (CR) and partial response (PR). LCR = (CR + PR + SD/total cases)%. The clinical efficacy evaluation of ^125^I brachytherapy was assessed by the Response Evaluation Criteria in Solid Tumors Version 1.1 (RECIST 1.1). The CBR was evaluated according to the PS score and pain score. The pain was evaluated by digital assessment (NRS) and positive CBR indicating an improvement in the patient’s functional impairment. PS score according to eastern cooperative oncology group (ECOG): 0-5. Upper limb edema was measured by measuring the circumcubitus 10 cm. OS was defined as the time between the date of ^125^I brachytherapy and the last follow-up or death. Adverse reactions were assessed according to the new classification of the Society of Interventional Radiology.

### Complications

The patients were observed for fever, bleeding, bone marrow suppression, liver and kidney dysfunction, radioactive skin and mucosa reaction, particle displacement, and other symptoms after seed implantation. The skin-mucosal response was assessed according to the Radiological Collaboration in Oncology/European Research and Treatment in Oncology (RTOC/EORTC) radiological injury grading criteria.

### Statistical analysis

The data analysis was performed using SPSS (version 24.0). Continuous variables were expressed as the mean ± SD. Paired t-test was used to analyze the scores of D90, V100, and V150 and pain and physical status before and after surgery. Comparisons of PS before and after the procedure were performed using the Mann–Whitney U test. Survival analysis was assessed by Kaplan–Meier methods. *p*< 0.05 was considered as statistical significance.

## Results

### ^125^I implantation and dosimetry description

A total of 15 patients received ^125^I seed implantation to meet the TPS criteria and followed by a postoperative dose evaluation. The seed activity was 0.3–0.7 mCi, and the median number of ^125^I seeds implanted was 33 (range: 20–130) with a median dose 90 (D90) of 108 Gy (range: 60–120 Gy) (**Table 2**). There was no significant difference in D90, V100, and V150 before and after operation (*p* > 0.05), as shown in **Table 3**.

**Table 2:** The information of 125I seed implantation.

NO	Activity (mCi)	Number of^125^I seed	*D*_90_ (Gy)	Pre/post-operative Max diameter at 6 months(cm)	Follow-up (months)	NRS(Pre/post- operation)	PS(Pre/post- operation)	Effect
1	0.7	130	108	10/6	7	5/1	3/1	PR
2	0.6	48	114	7/5.5	6	3/0	3/1	PR
3	0.5	33	120	3/0	9	0/0	1/0	CR
4	0.5	40	110	6/2	6	0/0	1/0	PD
5	0.7	70	120	9/2	8	4/1	2/1	PR
6	0.6	30	120	5/0	11	3/0	1/0	CR
7	0.6	59	110	12/5	15	3/1	2/1	PR
8	0.6	30	115	3/2	6	3/3	2/0	SD
9	0.6	20	100	3/2	6	1/0	0/0	SD
10	0.5	30	80	5.5/2	14	2/0	1/1	PR
11	0.5	50	80	3/2	11	4/3	2/1	SD
12	0.3	30	100	5/2	9.5	5/2	1/0	PR
13	0.3	30	60	3.3/2	18	1/0	1/0	SD
14	0.4	80	87	6/3	14	4/3	2/1	PR
15	0.5	30	80	4.5/2	6	5/2	1/1	PR

**Table 3:** Comparison of dose indexes in 15 patients ( $\bar{x}$ ± S).

	*D*_90_ (Gy)	*V*_100_ (%)	*V*_150_(%)
Pre-operation	103.58 ± 22.38	80.44 ± 13.084	46.86 ± 19.47
Post-operation	97.92 ± 26.01	69.01 ± 29.03	51.81 ± 15.78
*t*	1.143	1.644	1.157
*P*	0.179	0.122	0.267

### Clinical efficacy evaluation and overall survival

**Figure 1:**
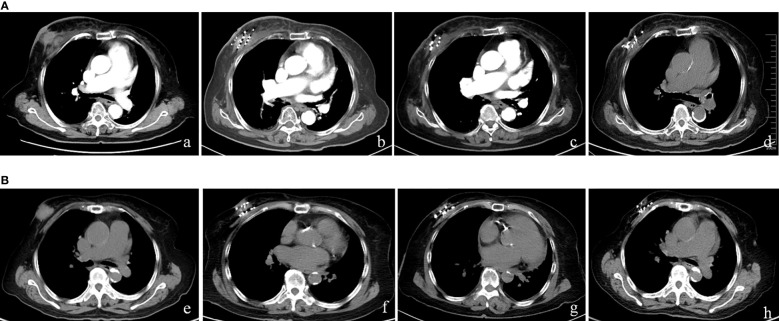
**(A)** An 89-year-old woman with the chest wall lesion recurred from right BC was accurately inserted into the tumor to implant 125I seeds. (a) Pre-brachytherapy; (b) 3 months after brachytherapy, tumor shrinked; (c) 5 months after brachytherapy, tumor disappeared; (d) 9 months after brachytherapy, no recurrence. **(B)** An 82-year-old woman with the chest wall lesion recurred from right BC. (e) Pre-brachytherapy; (f) 1 month after brachytherapy, tumor shrinked; (g) 11 months after brachytherapy, tumor disappeared; (h) 15 months after brachytherapy, no recurrence.

The patients were followed for 6–18 months (median: 9 months). Six months after operation, among the 15 patients, CT images showed that there were two cases of CR, eight cases of PR, four cases of SD, and one case of PD (**Figure 1**, CT images of 2 patients showed CR after 125I seed implantation therapy). The ORR was 66.7% (10/15), and the LCR was 93.3% (14/15) at 6 months. The maximum diameter of the target lesion decreased from (5.69 ± 2.69) cm before operation to (2.50 ± 1.74) cm after operation, and the difference was statistically significant (*p*< 0.05). At last of follow-up, five of these patients died of systemic metastasis and three of 15 patients died of organ failure. The 6- and 12-month OS were 100 and 41.6%, respectively, and the median survival time was 12.5 months.

**Figure 2:**
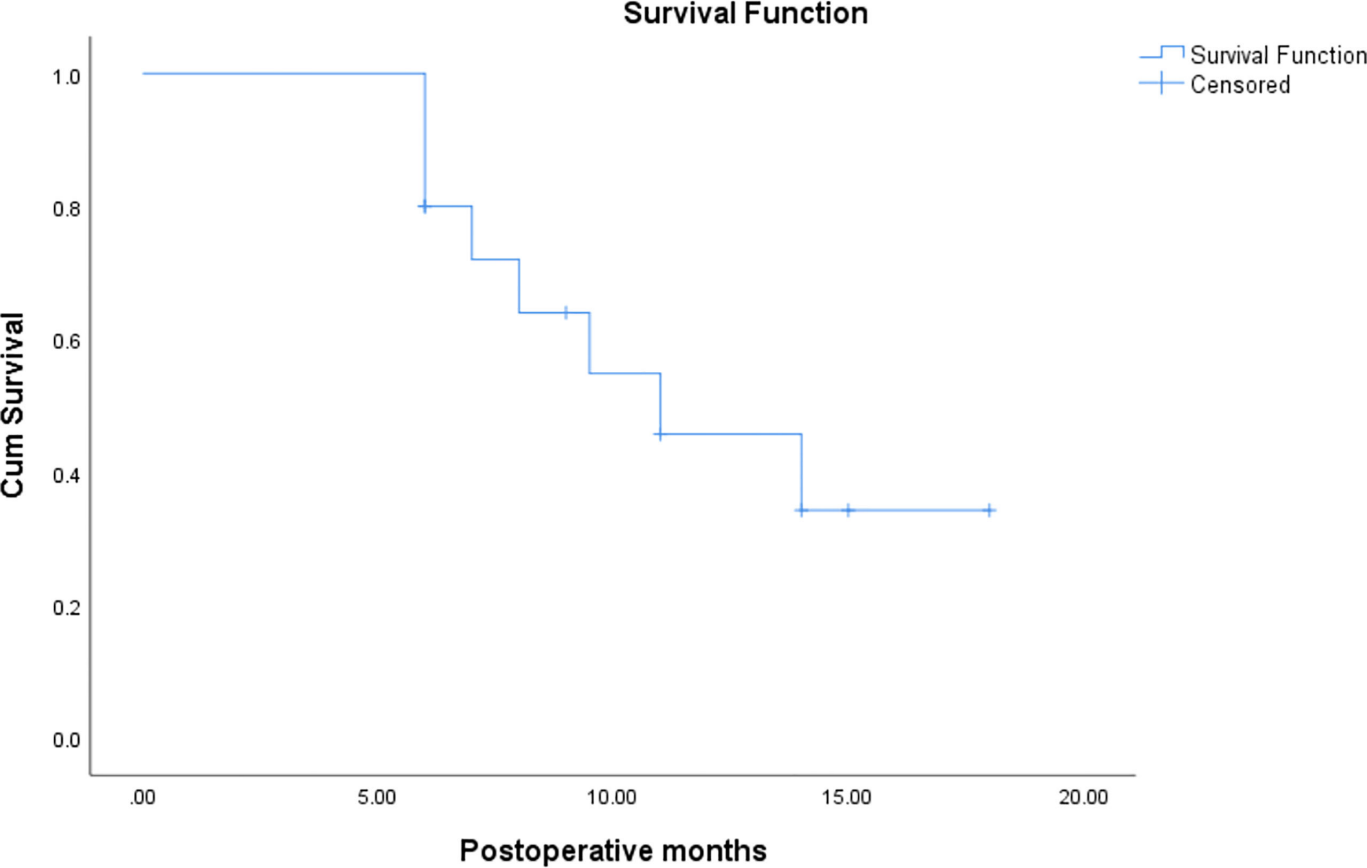
The survival rate of BC patients.

After operation, 11 patients received medicine therapy, including chemotherapy, endocrine and target, two cases showed CR, six cases with PR, three cases with SD, the ORR was 72.7%, LCR was 100%, the 6- and 12- month OS rate were 100 and 27.3%, respectively. While four patients just received single-radioactive seed implantation therapy, two cases showed PR, one with SD, one with PD, no case showed CR, ORR was 50%, LCR was 75%, and the 6- and 12-month OS were 75 and 25%, respectively. Survival analysis was shown in **Figure 2**. It seems that patients who received drug therapy after ^125^I seed implantation therapy has better local control and 6-OS.

### Clinical benefit response

We used PS and pain score to evaluate the CBR. PS score of these patients decreased from 1.53 ± 0.81 to 0.53 ± 0.49. The pain score decreased from 2.87 ± 1.67 before operation to 1.07 ± 1.18 after operation, and the differences were statistically significant (*p*< 0.05). The swelling of three cases of upper limb edema was gradually reduced within 1–14 days after operation, as shown in **Table 1**.

### Adverse events

Among these patients, five of 15 patients were elderly patients (older than 80 years), and six of 15 patients were complicated with chronic underlying diseases. There were no complications such as fever, hemorrhage, bone marrow suppression, liver and kidney dysfunction, radioactive skin and mucous membrane reaction, and particle displacement during follow-up period in all of these 15 patients. Furthermore, one patient with local huge ulcer at the recurrent lesion of the chest wall also has no radioactive skin injury after operation.

## Discussion

BC has been the most commonly diagnosed cancer worldwide and the leading cause of cancer death worldwide in women. For patients with local recurrence and metastasis, the overall prognosis is poor. Study showed that the majority of BC recurrences occur during the first decade after initial diagnosis with a peak incidence 2–5 years (12). However, the 10-year OS of patients with postoperative recurrence of BC was 39% and the distant disease-free survival rates was 36%, even after successful salvage mastectomy (13). The treatment for loco-regionally recurrent BC is challenging, especially the cases previously treated with multi-modality therapy. The most common therapies such as surgery, EBRT, chemotherapy, or hormonal interventions have limitations. Some large local-regional recurrent tumors cannot be completely removed. Chemotherapy and hormonal interventions failed to achieve therapeutic benefits; the second EBRT has certain curative effect, but with increasing toxicities (14). Therefore, another palliative therapy should be considered.

Brachytherapy demonstrates definite curative effect and few complications, which has been a safe, effective, and extremely versatile radiation technique (15). Meanwhile, existing studies have preliminarily confirmed that brachytherapy is also an ideal technique for residual disease and recurrences in BC patients based on its efficacy, appropriate toxicity, and cosmetic outcomes (15). ^125^I seed implantation is LDR brachytherapy for the release of low-energy γ-rays to kill tumor cells continuously; it is so safe with a sharp drop-fall off dose, and only 1.7-cm soft tissue could shield half the radiation dosage (16). To date, ^125^I brachytherapy is emerging as a salvage method for the treatment of many tumors, such as recurrent gliomas, retroperitoneal recurrent carcinoma, the head and neck, and recurrent ovarian cancer (17–20). In our center, ^125^I seed implantation is confirmed as a effective and safe therapy in most solid tumors, such as lung cancer (21), skin squamous cell carcinoma (22), malignant solitary fibrous tumor (23), and also as an effective salvage treatment for lymph metastases or loco-regional recurrence (24, 25). Furthermore, ^125^I seed implantation has become a promising new strategy for the treatment of cancer pain because of bone metastasis (26). However, there are few reports on local recurrence and metastasis BC patients, especially elderly patients with complications.

The current study preliminarily analyzed the clinical efficacy and safety of ^125^I seed implantation in the treatment of 15 cases of local recurrent metastatic BC patients. Results showed that the ORR and LCR of 6 months after ^125^I seed implantation therapy were 66.7 and 93.3%, respectively; the 6- and 12-month OS were 100 and 41.6%, respectively; and the median survival time was 12.5 months, no complications related to radioactive seed implantation occurred. These results were similar to Yu YH’study (27), who retrospectively analyzed 36 locoregionally recurrent and unresectable BCs to assess the efficacy of ^125^I seed implantation brachytherapy as a palliative management, results showed that 6-month local control was 97.2%, pain relief response rate was 88.9%, no serious complications were detected during the follow-up period, but 1-year OS was 97.2%, higher than that in our study (41.6%), and the reasons were analyzed that most of the BC patients in our study were elderly patients with underlying disease including heart disease, diabetes, and so forth. Furthermore, the diameter of tumor lesions were relatively large, the maximum diameter of tumors in this study was 12 cm, no tumors were less than 3cm, 60% of tumors were more than 5 cm, and last but not least, most patients had multiple systemic metastases; the systemic condition was poor. Some cases showed local tumor rupture accompanied by infection, so the prognosis was poor and the survival rate was relatively low. It was worth noting that we found that the pain score of BC patients decreased from 2.87 ± 1.67 before operation to 1.07 ± 1.18 after operation; PS score of these patients decreased from 1.53 ± 0.81 to 0.53 ± 0.49. It suggests that ^125^I brachytherapy is an effective method for the treatment of cancer pain. Furthermore, we also found that patients who received drug therapy after ^125^I seed implantation therapy seems has better local control and short survival, but it need larger cohort to confirm our findings.

To date, the dose and the activity choice of ^125^I seed implantation are controversial, no standard guidelines. Our previous study showed that ^125^I seed brachytherapy with a dose (EQD2) of 85-123.25Gy was safe for previously treated patients (23). In this study, the activity of^125^I seed implanted was 0.3–0.7 mCi, the median dose of ^125^I seeds implanted was 130 Gy (range: 110–160 Gy), and median dose of D90 was 97.92 ± 26.01 Gy (range: 80–120 Gy) after operation; there was no significant complications. Yu YH et al (26)reported the median activity of ^125^I seed was 0.6 mCi (range: 0.4–0.7mCi), which was similar to our study, but MPD was 110Gy (range: 90–140 Gy), lower than that in our center. The experience of our center was based on the location, volume, and distance between the target area and the surrounding organs: First, some patients had received external radiotherapy and their overall condition was poor, the prescription dose was reduced, and the irradiation in the high-dose area was reduced. For example, the D90 of ulcerative cancer focus of case no. 13 was 60 Gy. On the one hand, due to the patient’s previous external radiotherapy, the tumor was large and the surface skin was ruptured and infected. The target area of this case was zero distance from the skin of the organ at risk, and we selected the particle activity with 0.3 mCi. The ulcer was reduced and stable after surgery, and no complications occurred. Second, based on the pathological type, BC is moderately sensitive to radiation, and prescription dose should be reduced.

This study has several limitations. First, it is a retrospective study; prospective multi-center study is needed in the future to avoid the bias. Second, the number of patients is relatively small; a larger cohort should be conducted in the future to confirm our findings. Finally, this is a single-arm study, no parallel controls, and only external historical data can be compared with evaluate the safety and efficacy of this method. It is difficult to obtain historical research data that are completely consistent with the current study design, resulting in a bias in evaluating the results.

In conclusion, CT-guided ^125^I seed implantation brachytherapy is a safe and effective radiation technique for recurrent BC with definite curative local therapeutic effect and safety. This therapy may become a promising new strategy for local-regional recurrent BC patients, especially for elderly patients with complications who are not eligible for surgical resection and/or fail to benefit from EBRT and systemic therapy.

## Data availability statement

The raw data supporting the conclusions of this article will be made available by the authors, without undue reservation.

## Ethics statement

The studies involving human participants were reviewed and approved by the institutional review board of Hebei General Hospital. The patients/participants provided their written informed consent to participate in this study. Written informed consent was obtained from the individual(s) for the publication of any potentially identifiable images or data included in this article.

## Author contributions

HZ was responsible for the overall design of this study. JW and XC was responsible for manuscript writing as well as data analysis. KX, YL JZ and ZL were responsible for data collection. All the authors reviewed the manuscript. All authors contributed to the article and approved the submitted version.
